# 1803. Analysis of National Policies and Antimicrobial Resistance Burden: An Ecological Study of 114 Countries

**DOI:** 10.1093/ofid/ofad500.1632

**Published:** 2023-11-27

**Authors:** Moises R Vargas, Kevin Ikuta

**Affiliations:** University of California Los Angeles, Los Angeles, California; West Los Angeles VA, Los Angeles, California

## Abstract

**Background:**

The rise of antimicrobial resistance (AMR) is a top public health concern. The drivers of AMR are complex, with antimicrobial misuse, lack of coordinated policies and inter-country data cooperation playing key roles. The growing burden of AMR is not shared proportionally across countries and there’s marked heterogeneity of national-level policies to tackle AMR. Our aim was to evaluate whether stronger national policies correlate with reduced burden 3rd generation cephalosporin (3GC) resistant *Escherichia coli* utilizing two recently published estimates.

**Methods:**

We used 2019 estimates of deaths and Disability Adjusted Life Years (DALYs) due to 3GC resistant *E. coli* published by Murray et al. We matched these estimates to the governance score produced by Patel et al, which analyzed National Action Plans (NAP) through a governance framework, assigning a “governance score” to countries based on 54 indicators across 3 domains (Table 1). We describe the relationship between the burden of 3GC resistant *E. coli* and governance score across six WHO regions: Africa, Americas, Eastern Mediterranean, Europe, South-East Asia, and Western Pacific.

**Figure:**
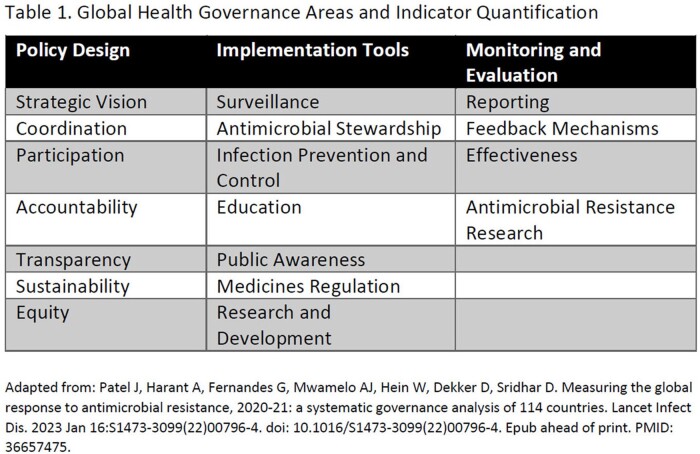

**Results:**

A total of 114 countries with both governance score and 3GC resistant *E. coli* data available were included. A multivariate log-linear regression model adjusted for region showed a relative risk reduction in the mortality of 3GC resistant *E. coli* of 2.8% (CI 1% –4.6%; p-value 0.002) for every increase in point of governance score (Figure 1).

When looking at DALYs as a measure of AMR burden, using a multivariate log-linear regression model adjusted for regions, we find a relative risk reduction of 4.7% (CI 1.8% - 6.2%; P value < 0.001) for every increase in point of governance score (Figure 2).

**Figure:**
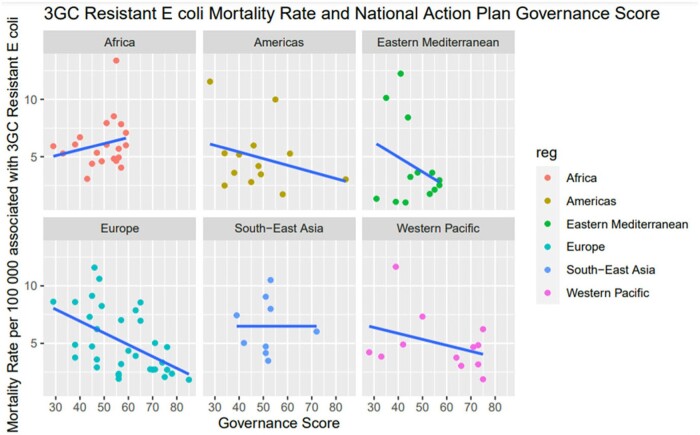

**Figure:**
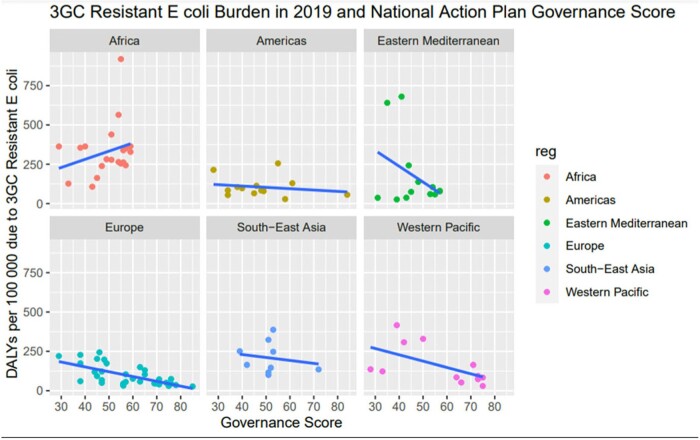

**Conclusion:**

We show that countries with high governance scores had overall lower burden of 3GC resistant *E. coli.* Additional time series analysis as well as evaluating specific aspects of NAPs against AMR burden would be an important next step.

**Disclosures:**

**All Authors**: No reported disclosures

